# There is no free benchmark: An institutional view of legal AI benchmarking

**DOI:** 10.1073/pnas.2509757122

**Published:** 2026-07-20

**Authors:** Neel Guha, Andy K. Zhang, Christine Tsang, Christopher D. Manning, Julian Nyarko, Daniel E. Ho

**Affiliations:** ^a^ Stanford Law School, Stanford University, Stanford, CA 94305; ^b^ Department of Computer Science, Stanford University, Stanford, CA 94305; ^c^ Regulation, Evaluation, and Governance Lab (RegLab), Stanford University, Stanford, CA 94305; ^d^ Department of Electrical Engineering and Computer Sciences, University of California, Berkeley, CA 94720; ^e^ Department of Linguistics, Stanford University, Stanford, CA 94305; ^f^ Stanford Institute for Human-Centered Artificial Intelligence (HAI), Stanford University, Stanford, CA 94305; ^g^ Legal Innovation through Frontier Technology Lab (liftlab), Stanford University, Stanford, CA 94305; ^h^ Department of Political Science, Stanford University, Stanford, CA 94305; ^i^ Stanford Institute for Economic Policy Research (SIEPR), Stanford University, Stanford, CA 94305

**Keywords:** AI, legal technology, institutional design

## Abstract

Despite substantial excitement around the use of AI in law, little information exists on the performance and associated risks of the domain’s widely marketed tools. Recent work, for instance, has demonstrated the significant potential for “hallucinations”—wherein models make up facts, law, and precedent—leading Chief Justice Roberts to spotlight this risk in his annual report on the judiciary. We argue that there is a need for public AI benchmarking in law. First, relative to other AI application domains, the legal AI ecosystem lacks legibility—there is little information about the design and performance of many commercial legal AI systems. Legal AI has not benefited from the types of benchmarking that have catalyzed, measured, and informed AI innovation and responsible use in other domains. Second, we articulate the challenges of the institutional design of benchmarking. We illustrate how benchmarks can be captured, watered down, and abused. Careful institutional design around the why, who, what, and how of benchmarking will be critical to navigate difficult tradeoffs of transparency, objectivity, expertise, and resources. Third, addressing legal AI’s illegibility requires matching institutional models to available resources and constraints. Rather than advocating for a single “best” approach to benchmarking, we show how benchmarking strategies depend on available resources.

Excitement about the opportunities enabled by AI—and most notably, generative AI systems—has sparked substantial investment and development across a wide range of domains (1). Amid this activity, the law has become something of a poster child for AI’s potential, with performance on legal tasks used to illustrate AI advances (2). Indeed, modern large language models’ (LLMs) text generation and reasoning capabilities appear tailor-made for the law’s distinctive technical challenges. AI offers the promise of cheaper, more efficient, and more accessible legal services (3). In the wake of ChatGPT’s release, there has been a flurry of new AI systems for contract review and analysis, legal research, litigation, compliance monitoring, e-discovery, and workflow management (4).

At the same time, enthusiasm for AI is dampened by pragmatic concerns about performance (5). A growing evidence base suggests that building useful legal AI tools is hard and that systems can fail in critical ways. Prior work has documented instances of hallucination (6, 7), susceptibility to bias (8), and uneven performance across different areas of the law (9). Some failures are conspicuous—such as the more than 120 documented cases involving AI-fabricated citations and law (10, 11)—while others are subtler, arising when tools misinterpret legal arguments or cite sources for incorrect propositions (6, 12). A volatile public conversation seesawing between legal AI’s promise and flaws has produced substantial uncertainty as to where tools can be trusted for use today, and where additional development is needed (13).

In other domains, institutional stakeholders have approached similar concerns through community-driven “benchmarking” processes, in which multidisciplinary expert groups assess the performance of AI systems and forecast their impact (14). Sometimes referred to as “evaluation,” the process of benchmarking is a science and engineering discipline unto itself, with specialized practices, tools, and methodologies (15). From medicine to software engineering to facial recognition, information from benchmarking has aided risk/benefit assessments and guided responsible adoption (16–18).

This paper argues that realizing AI’s promise for the law requires greater public benchmarking, on par with that of other domains. Benchmarking is necessary because legal AI today lacks legibility. That is to say, there is little public information about the performance of commonly used legal AI systems. Legal AI’s illegibility threatens responsible deployment, stymies efforts to legal education efforts, slows innovation, and results in poorer governance of the technology.

Prior work has approached legal AI benchmarking from a technical perspective, and conducted evaluations of specific systems, built benchmarks, and proposed evaluation methodologies (3, 6, 19, 20). In contrast, we offer an institutional perspective (21, 22). Examining legal benchmarking through an institutional lens calls attention to a different set of considerations, around benchmark ownership, incentives, and capacity (23). The justifications for an institutional analysis of benchmarking are twofold. First, benchmarking is a highly discretionary process in which the responsible entities make choices that shape both the quality and type of information produced. Second, the characteristics of the institutions conducting benchmarking—such as their incentives, capabilities, and position within the broader ecosystem—inevitably influence these choices. Accordingly, an institutional analysis can 1) anticipate what kinds of information a given institutional arrangement is likely to generate, and 2) ask what institutional design is needed to produce a desired distribution of benchmarking information.

Our institutional focus reflects a necessary paradigm shift. Until recently, much of legal AI benchmarking has occurred within academic settings, with computer science and legal scholars focused on understanding whether computational systems can perform “legal reasoning” (3, 20, 24–26). In this context, institutional questions hold less relevance. But as legal AI moves from research prototypes to deployed systems, questions of who funds, participates in, and organizes benchmarking grow more relevant to its practical exercise.

Our paper makes three contributions. First, we explicate the core design choices involved in benchmarking (Section 1). Without considering the tradeoffs of these choices, benchmarks can be poorly implemented, gamed, and manipulated. Second, we conduct an institutional analysis of legal AI, identifying how different stakeholders—such as legal AI developers, purchasers, academics, bar associations, courts, and governmental oversight bodies—may approach these different choices (Section 2). Our findings, combined with other observations about the lack of public performance information in legal AI, lead us to conclude that legal AI suffers from illegibility. Third, we propose that addressing legal AI’s illegibility requires matching institutional models to available resources and constraints (Section 3). Our analysis considers three institutional models, each suited to a different level of resource availability: National Institute of Standards and Technology’s (NIST) Face Recognition Vendor Test (FRVT) exemplifies high-resource government coordination; industry consortia illustrate medium-resource private leadership; and discrete academic grants demonstrate low-resource targeted research. We assess how each addresses the institutional tradeoffs identified under distinct resource conditions.

## 1. Benchmarking’s Design Dimensions

We first explain the traditional benchmarking process and identify its “design space:” six core decisions that shape the information it produces. We then explain why benchmarking matters, compare the information produced by benchmarking to other kinds of information about AI, and formalize the notion of “legibility.”

### 1.1. Benchmarking’s Design Dimensions.

Benchmarking is the process by which stakeholders measure the performance and impact of AI technologies (15). It can target different levels of analysis: the AI system (e.g., ref. 6), individual machine learning models within the system (e.g., ref. 14), or the combination of a human working with the system (27). The kind, quality, and utility of information produced depends on how benchmarking is structured. We refer to the key choices involved as benchmarking’s six “design dimensions,” and review each in turn.

#### 1.1.1. Task definition.

Benchmarking begins with determining the exact task to be evaluated. In legal AI, examples of tasks include summarizing judicial opinions, producing redlines of contracts, classifying the enforceability of lease provisions, or answering open-ended research questions. Task definition sets the boundaries of what a benchmark can meaningfully evaluate, as a benchmark can only generate insights about the tasks it explicitly includes. Selecting those tasks requires attention to users’ informational needs and the range of real-world applications. This challenge is magnified by newer “general-purpose” legal AI tools designed to support a wide array of legal activities. If a benchmark defines tasks too narrowly—focusing, for instance, only on summarization—it may overlook performance on other vital functions such as legal reasoning, document drafting, or verifying the precedential value of a case. Worse, such narrow benchmarks may mislead stakeholders by selectively highlighting favorable capabilities while ignoring areas of weakness. To be useful, benchmarks must reflect the breadth of actual usage and anticipate the diversity of legal contexts in which AI systems operate.

#### 1.1.2. Benchmark construction.

The second consideration is how benchmark data is constructed. Once a task is defined, what specific data samples should systems be evaluated on? If the task is determining the enforceability of leases, construction entails selecting the lease provisions to be analyzed. If the task involves answering legal research questions, it requires crafting representative and meaningful queries. In all cases, benchmark construction involves a series of task-specific discretionary choices.

Construction decisions shape benchmark difficulty (28), and two benchmarks for the same task definition might yield sharply different performance estimates depending on the distribution of the underlying samples. Construction decisions also affect the external validity of a benchmarking process (29, 30), and a benchmark whose data do not match the real world will yield misleading performance estimates (31). Constructing a useful benchmark thus requires a clear understanding of real-world usage contexts and the types of inputs systems routinely encounter.

Two examples illustrate these points. First, an academic study found that a Thomson Reuters AI-based legal research tool—touted as “avoiding” hallucinations—hallucinated in 33% of benchmark queries (6). In response, Thomson Reuters provided, for the first time, its own internal measurement of error rate of 10% to assert that internal tests differed dramatically (32). Differences in benchmark construction and their associated questions may explain this discrepancy. The academic study made such information available, but without understanding the company’s benchmark construction, it is impossible to know.

A second illustration comes from Paxton AI issuing a press release with the headline “Paxton AI achieves 94%+ accuracy on Stanford Hallucination Benchmark.” The task was a tool to classify whether cases are positively or negatively associated based on the citation. An earlier academic study of vendors found significant rates of disagreement and “egregious errors” across products (33). Paxton, however, evaluated on a benchmark dataset that is so easy (whether a case explicitly overruled another case) (24) that even simple bag-of-words models achieve near-perfect performance.

#### 1.1.3. Choice of evaluation protocol.

The third consideration is how systems are evaluated on a benchmark (the evaluation protocol). Generative AI systems in particular can be applied to the same benchmark in multiple ways. Because such models often produce different outputs for identical prompts, benchmark designers face a host of decisions. Should systems be run multiple times per input? If so, should evaluations consider the best, worst, or average output? Similarly, many systems allow customization through adjustable parameters or settings. Benchmarking must therefore standardize these configurations to ensure fair comparisons.

Seemingly minor adjustments to evaluation protocols can yield substantial differences in reported performance. Across domains, AI researchers have observed how inconsistent evaluation practices prevent accurate comparisons, with methods appearing to outperform others simply due to favorable evaluation setups (34, 35). Standardized protocols can reveal that purported improvements vanish or that competing systems perform roughly equally. A striking example came when Google used a nonstandard prompting technique to boost the reported performance of its Gemini model on the MMLU benchmark (36), allowing the company to claim that Gemini outperformed OpenAI’s GPT-4. Yet when both models were reevaluated under standardized conditions, Gemini’s reported advantage disappeared and GPT-4 ultimately performed better.

#### 1.1.4. Choice of measurements.

A fourth consideration is the type of measurement that benchmarking efforts produce. One key dimension concerns subgroup performance. A legal research tool, for instance, might be evaluated across question types or areas of law, while a lease analysis tool might be assessed by geography, jurisdiction, or lease characteristics (9). Such disaggregation is critical: Decisions about whether an AI system can be relied upon can depend on worst-case outcomes just as much as average performance (37). Researchers studying medical AI have demonstrated this principle through examples of “hidden stratification,” where models that appear to perform well overall can fail badly on clinically important subgroups. In one case, a chest X-ray model achieved an ROC AUC of 0.87 for pneumothorax detection overall, but only 0.77 for the most clinically critical cases—patients without chest drains, whose pneumothorax remains untreated and life-threatening (38). In an industry context, it is common for sophisticated AI teams to devote substantial resources toward subgroup selection and management (37).

Benchmarking also requires deciding what aspects of performance to assess. This depends on context-specific values and use cases. For example, a chatbot assisting tenants facing eviction should be evaluated not only on correctness but also on tone, clarity, and emotional appropriateness—since flippant responses during a housing crisis would be unacceptable. A challenge in legal AI is that many tasks lack measurement or quality standards. For example, there is no universally accepted metric for what makes a contract “good,” or for whether a legal summary captures the right nuance. Benchmarking in these instances must navigate both normative uncertainty and practical ambiguity about what constitutes success. Two benchmarking processes might yield different results because of subjective disagreements regarding AI output quality.

#### 1.1.5. Choice of systems.

A fifth choice concerns which systems the benchmarking effort evaluates. This includes not only which vendors are covered but also which of a vendor’s offerings are selected, since many companies provide multiple tools with different performance and cost tradeoffs.

For evaluation of generative AI tools, benchmarking costs can scale directly with the number of systems being evaluated. Today, benchmarking for generative AI outputs in domains like law typically require manual evaluation.^*^ But robust manual evaluation can be very expensive and time-consuming. Multiple trained annotators are often needed to resolve disagreements, and each output must be carefully scrutinized as generative AI mistakes can be subtle.

There is no obvious answer for how to navigate this challenge or select which systems to include. For example, a simple cost-control approach like evaluating only the most widely used tools has its own downsides. It reduces smaller companies’ ability to compete by denying them opportunities to demonstrate superior performance relative to larger players. It also entrenches established companies, who can leverage both the benchmarking information for technical development and their participation for marketing advantages. Finally, users of smaller tools receive no performance information about the systems they actually use. Unfortunately, benchmarking may be most expensive precisely where it is most helpful—in markets with many seemingly indistinguishable products.

The perils of system selection are illustrated by Chatbot Arena (39), a widely used benchmark for chatbot evaluation. Chatbot Arena scores LLMs through head-to-head battles where users submit prompts and assess which model produces the better response. This user feedback generates a competitive leaderboard that LLM companies frequently cite in their marketing, making rankings extremely valuable economically.

However, a recent analysis revealed that undisclosed policies granted disproportionate access to a handful of “preferred providers,” allowing them to privately test many model variants and publicly release only their best-performing versions (40). Additional access allows developers to “game” the leaderboard and artificially boost their ranking. Indeed, the model version Meta used for Chatbot Arena was different from the one they publicly released (41). The evaluation structure also created disparities in human feedback data, with commercial models receiving more feedback than open-source alternatives. Since more data allow developers to tailor model performance in Arena-specific ways, this created additional unfair advantages. These issues illustrate how the seemingly straightforward question of which systems get benchmarked can profoundly complicate efforts to create fair and useful evaluations.

#### 1.1.6. Transparency.

The final consideration pertains to the transparency of the benchmarking process. Transparency exists on a spectrum: At one end, organizers release all data and code; at the other, they share only results and broad descriptions. Between lie intermediate approaches, such as publishing some tasks but not others, or releasing small representative subsets.

Transparency implicates major tradeoffs. Greater openness builds trust in the benchmarking process among stakeholders, enables external review, and spurs innovation in both the systems being evaluated and the evaluation methods themselves. Public benchmarks have long undergone iterative improvement, as researchers refine and expand them after uncovering errors or limitations (42).

Yet transparency also creates risks of gaming that can erode the validity of the benchmark. Once datasets are public, developers may intentionally or unintentionally train on them, meaning that results may reflect model memorization rather than genuine performance. An organization which chooses to release benchmarks cannot reuse them. The need to develop wholly new ones for each benchmarking effort significantly raises costs.

Gaming concerns are widespread and well-founded. AI companies have been accused of behavior that is in the best case opportunistic and in the worst case fraudulent (43, 44). Even inadvertent contamination poses serious risks, and studies have identified instances where previously reliable benchmarks became unusable due to train-test leakage that occurred when benchmark data was posted online and subsequently incorporated into web-scraped training datasets (45, 46).

### 1.2. Public Benchmarking and Legibility.

We describe a product ecosystem surrounding a particular domain as “legible” if it provides public benchmarking information about the different systems used in that domain. Illegibility can arise because public information is insufficient, inaccurate, or unrepresentative. Illegibility can be contrasted with other informational problems often associated with AI. For example, researchers and policymakers often discuss problems with AI opacity, which usually relates to the fact that AI systems may be uninterpretable (i.e., we do not know what internal rules they follow) or unexplainable (i.e., we do not know why specific predictions were produced). Illegibility may also be contrasted with a lack of developer transparency. This is often used to describe the fact that we do not know how developers build systems, what objectives they have in mind, where systems are used, or the kinds of internal validation and quality assurance that systems are subject to.

Legibility requires publicly available benchmarking information about the commonly used systems in a particular domain. Illegibility is also partially dependent on the technical sophistication of individuals within an ecosystem, and their capacity to understand benchmarking information. Importantly, this means legibility is more likely to exist in communities who are deeply experienced in using AI.

Illegibility demands special attention because benchmarking information functions as a sort of “engine oil” necessary for various forms of decision-making.^†^ Illegibility also matters since the other kinds of information discussed above are poor substitutes. Benchmarking is essential given that modern AI models may contain billions of parameters, making it nearly impossible to predict their behavior based solely on their internal structure. Moreover, it is difficult to anticipate how humans will interact with or respond to these systems in practice. Benchmarking addresses this uncertainty by providing empirical insight: Through systematic observation of input–output behavior, it enables practitioners to forecast how systems will perform and what effects they may have in real-world settings. The need for benchmarking is analogous to the role of human testing in drug development. Just as we cannot determine a drug’s safety or efficacy purely from its molecular composition, we usually cannot fully evaluate an AI system without empirical testing on benchmarks.

## 2. Legal AI’s Illegibility

This section applies the institutional lens to identify and explain legal AI’s illegibility. It first describes the ways in which legal AI is illegible, drawing specific observations about the lack of public performance information and public benchmarks. It then uses the benchmarking framework offered in Section 1 to examine whether different actors within legal AI can supply public benchmarking. For each potential actor, it asks how their incentive structures, relative access, and competencies affect the benchmarking design choices they might make, and thus, what kinds of public benchmarking information they might produce. Overall, this analysis reveals a fundamental tension: Institutions with the resources and access needed for comprehensive benchmarking face systematic conflicts of interest, while those whose independence might be trusted lack the technical expertise or financial capacity to execute effectively. Table 1 summarizes these institutional tradeoffs across benchmarking design dimensions. Finally, this section closes by identifying the implications of legal AI’s illegibility, for education, innovation, governance, and more.

**Table 1.** Institutional competency across benchmarking design dimensions

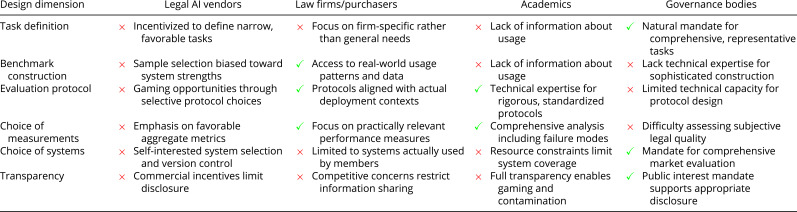

### 2.1. The Case for Legal AI’s Illegibility.

Even as commercial AI systems proliferate across legal practice, little is known about their risks and performance. Consider research systems based on large language models, such as those offered by Westlaw, LexisNexis, and newer companies like Harvey (6). These tools are intended to replace or augment traditional keyword-based research with conversational interfaces that generate legal answers. Yet there is little public information on how often these systems produce hallucinations, decline to answer, reaffirm incorrect assumptions, or simply fail to provide helpful output. Nor is there clarity on how such behaviors vary across question types, legal domains (e.g., securities law vs. criminal procedure), or source types (e.g., judicial opinions vs. statutes). While a small body of academic work has begun to explore these questions (6), the rapid pace of model development and the number of available tools make it difficult to keep evaluations current or comprehensive.

The lack of information also extends to how humans interact with legal AI. Because many tools are designed to support and not replace legal professionals, overall effectiveness often hinges on user behavior. Elsewhere, researchers have shown that human factors surrounding an AI system influence its real-world outcomes (47, 48). A system that is accurate in isolation may deliver little real-world value if users spend excessive time second-guessing outputs. Yet beyond a handful of studies (49–51) and anecdotal reporting, we generally know little about how legal professionals actually use AI tools in practice, or how human–AI collaboration affects accuracy, efficiency, and error.

Legal AI also lacks both formal and informal standards for performance disclosure. Developers often claim to conduct internal benchmarking but provide little detail about the underlying data, metrics, or methods (6, 52). As a result, even when performance figures are shared, downstream users may struggle to interpret them meaningfully. Compounding this is the relative scarcity of sophisticated purchasers in the legal sector who can critically assess such information. This is a sharp contrast to fields like medicine, where hospitals have advanced IT departments, or general-purpose AI, where application developers invest heavily in evaluation.

Examining the ecosystem of legal benchmarks emphasizes these concerns. The ecosystem remains small, with few public legal AI benchmarks. Importantly, those that do exist suffer significant limitations. Many repurpose legal datasets in ways that do not closely mirror real-world legal tasks (19, 20, 24). Others focus narrowly on assessing the “reasoning” capabilities of large language models in isolation (3). With the notable exception of e-discovery (53), no public benchmarks currently exist for vast segments of practical legal work, like drafting briefs, redlining contracts, or answering legal research questions.

In other fields, by contrast, benchmarking is both prolific and institutionalized. There are benchmarks for a wide range of tasks in medicine (54), software engineering (16), security (55), and beyond. Top academic venues like NeurIPS host dedicated benchmark tracks to foster development (56). Independent research labs maintain leaderboards to track system performance over time (14, 57). And AI companies themselves frequently release benchmarks, recognizing their value in guiding innovation and shaping standards (58, 59). Legal AI remains an outlier in this regard—both under-benchmarked and under-studied.

Several considerations unique to legal AI make benchmarking quite challenging, and thus deserve mention. First, legal AI tasks are often technically harder to benchmark. Relative to other domains, legal AI has more generative tasks for which automated evaluation methods are not readily available (15). Generative tasks for other application areas have benefited from reliable automated methods, like unit tests and LLM-based evaluations (39, 60). Legal AI benchmarking efforts must rely on manual validation of system outputs to assess quality (6, 25, 33, 61). This makes benchmarking costly, as trained legal annotators are expensive. It also reduces the number of systems any benchmarking effort may assess, and how often benchmarking can occur. Legal AI also has more tasks which are “subjective,” and for which quality may be contested (15). The persuasiveness of a brief, for instance, may depend on the counterparty and judge (62). Aside from presenting tricky conceptual questions as to how evaluation should be structured, benchmarking thus requires more annotators to appropriately average over individual subjective preferences (63).

Second, legal tasks are also context-sensitive to a greater degree than other domains. In medicine, for instance, whether or not a patient’s chest X-ray indicates pneumonia does not change if the patient is in Palo Alto or Tuscaloosa. Contrast this with a task like answering questions about eviction processes. Evictions are governed by a mixture of both state and local law, which means the same questions might have different correct answers across counties. Answers might also change over time, based on updates to the law (through statutes, regulations, or case law). Meaningfully assessing a legal AI system’s capacity to perform this task would require benchmarking for potentially every county, every year.

Third and finally, the legal field has shown comparatively little emphasis on assessing the quality of outcomes or decision-making (64, 65). Disciplines like medicine have institutionalized evidence-based practices, underpinned by rigorous measurement systems (66). In contrast, legal metrics often focus on process and effort—such as time spent—rather than substantive performance or impact.

### 2.2. Institutional Analysis.

We now assess who within the legal AI ecosystem could theoretically supply public benchmarking.

#### 2.2.1. Legal AI vendors.

Legal AI developers could supply public benchmarking information by funding or producing public benchmarks, working through industry consortia to set up benchmarking efforts, or releasing information produced through internal benchmarking. While legal AI vendors do often have the resources and technical expertise that benchmarking requires, they face a number of other important limitations.

First, legal AI vendors’ incentives to produce fulsome and accurate public benchmarking information are murky. Vendors may still have reason to share performance information, particularly when doing so highlights their advantages over competitors. In principle, releasing public benchmarks might also yield reputational benefits and help steer academic research toward problems aligned with vendor goals (58).

In practice, however, vendors are primarily motivated by-product sales, and thus incentivized to design benchmarks that emphasize the comparative strengths or reliability of their systems. The flexibility of benchmarking’s design space provides many opportunities to tilt the process in their favor. Vendors might define tasks narrowly around areas where their systems perform well, choose sample distributions that accentuate their results, adopt evaluation protocols that showcase their capabilities, and stress aggregate performance metrics while avoiding subgroup analyses that could expose systematic weaknesses. With access to the benchmark, they may also engage in selective version control, optimizing models for benchmark performance while withholding less favorable results.

Examples of this kind of behavior are frequent in accounts of corporate misconduct or deception. Volkswagen famously relied upon “defeat devices” to evade emissions testing. And as the financial stakes surrounding AI continue to grow, similar concerns have begun to surface. Developers have faced criticism for tailoring systems to perform well on particular benchmarks and selectively reporting results (36). Absent independent oversight and scrutiny, ascertaining benchmark gaming is difficult.

Second, vendors may still lack information about how tools are (or will be) used, preventing them from designing representative benchmarks. Confidentiality and data secrecy requirements mean that vendors may lack the ability to observe how different clients use that tool. This constraint particularly affects benchmark construction, as vendor-produced benchmarks will constitute approximations of how they think their tools are used, rather than reflecting actual usage patterns. Whether or not these approximations are in fact accurate is, of course, an entirely separate matter which may vary across applications.

Some of these problems could, in principle, be mitigated through regulation. For example, regulators might require vendors to disclose results from internal benchmarking—where development needs create strong incentives for accuracy—or to conduct specific evaluations as a condition for marketing or selling their tools. Yet legal AI currently lacks this kind of product-centered governance. Much of the emerging regulatory focus has instead centered on uses of AI, and thus on the legal practitioners and institutions deploying these systems. As a result, few rules apply directly to the products themselves, leaving vendor benchmarking largely self-policed.

#### 2.2.2. Legal tech purchasers.

A second option is for purchasers of legal AI tools (e.g., law firms, in-house counsel) to lead public benchmarking efforts.

A central challenge here is the risk of rent-seeking. Because purchasers are often in competition with one another, they have strong incentives to treat benchmarking information as a proprietary asset. For instance, if two law firms stand to benefit from similar AI tools, it is likely they are competing for similar kinds of legal work and thus for the same clients. In this setting, access to superior benchmarking information can offer a competitive edge. A firm that identifies more effective or cost-efficient tools stands to extract greater value than its peers. This makes benchmarking knowledge a valuable commodity, and in turn creates powerful disincentives for firms to share that information openly, directly compromising the transparency needed for effective public benchmarking.

One way to mitigate this inefficiency is through coordination, such as by benchmarking consortia. Such consortia would allow legal technology purchasers to pool resources, share the costs of evaluation, and reduce redundancies from duplicative individual efforts. They might also yield better evaluations, drawing on diverse perspectives about how benchmarks should be designed. From a technical perspective, consortia also enable shared infrastructure, such as common hardware, repositories, and evaluation frameworks. Importantly, a consortium’s members would bring valuable insights about real-world usage patterns for benchmark construction and could design evaluation protocols that reflect actual deployment contexts rather than laboratory conditions.

However, this cooperative model raises its own set of distributional challenges. The legal services market is highly heterogeneous, and potential consortium members differ widely in size, resources, expertise, and technological capacity. Consider the range of prospective users of legal research tools: Global law firms with dedicated technical teams, solo practitioners, boutique litigation firms, in-house counsel, nonprofits, government lawyers, and public interest groups. These actors have starkly different abilities to contribute time, money, or technical infrastructure to a consortium.

These disparities must manifest somewhere in the structure of the consortium. One concerning possibility is self-segregation, with distinct consortia for different tiers of consumers. This would produce an ecosystem of “rich” and “poor” consortia, with resource-rich members generating higher-quality benchmarks and reaping disproportionate benefits. Another possibility is differentiated access within a single consortium. Members who contribute more (in capital, compute, or expertise) might receive more or better information, or enjoy greater influence over benchmarking design. In this case, we should expect benchmarking outcomes to reflect the interests and priorities of the largest firms. Task definitions and measurement choices would likely center on tools used in high-volume transactional practice, marginalizing use cases important to public interest groups or solo lawyers. Moreover, system selection would be limited to tools actually used by consortium members, potentially excluding innovative solutions that serve underrepresented market segments.

#### 2.2.3. Academics and independent groups.

Another option would be for academics or independent advocacy groups to drive public benchmarking, much like they already do for general domain AI. Universities with both law schools and computer science departments could assemble interdisciplinary expert teams capable of performing legal benchmarking. Legal nonprofit groups—like the Legal Services Corporation—have public interest missions, and so are amenable to both distributing benchmarking information and ensuring it is representative of diverse interests. They also lack the financial stake that legal technology developers or legal technology consumers often have, and so may be trusted to maintain objectivity.

The academic-centric approach, however, faces several challenges across multiple design dimensions. First, academics and independent groups may lack the resources necessary to produce comprehensive benchmarking. This problem will only grow worse in the face of cuts to federal research grants. The high cost of legal benchmark construction means that existing academic benchmarks often rely on clever repurposing of existing data, collaborative models to offset costs, or are extremely small in size (3, 24). Such approaches may mitigate costs, but often yield benchmarks that are narrower in scope, less representative, and difficult to generalize beyond their original data sources.

Second, publication guidelines typically require academics to make benchmarks fully accessible online for others to use, creating fundamental tensions around transparency choices. As discussed above, public benchmarks have limited utility in the longer term, due to the risks of intentional or accidental train-test contamination.

Third, academics may at times lack information about how legal AI tools are actually used in practice, making it challenging to define tasks and construct benchmarks that match real-world settings. Unlike law firms and developers, academics typically cannot observe authentic usage patterns or understand which performance characteristics matter most to practitioners. Attorney-client privilege and associated rules governing confidentiality prevent sharing of much real-world legal data (67).

Fourth, academics often lack the access necessary to engage in benchmarking. Most legal AI systems are not publicly accessible, and only available to institutional consumers like law firms. In contrast to domains where AI systems are available through APIs, academic researchers cannot easily perform independent benchmarking or verify developer-reported results (68). Additionally, the kinds of legal AI data necessary for benchmarking—case law, statutes, client memoranda, and more—are also not easily accessible to academics. Important institutional legal data are often surprisingly privatized, unpublished, or inaccessible (69). Legal search in the United States offers a useful example (70, 71). Comprehensive access to case law, statutes, and regulations is available through one of a small number of legal search companies. The law may be public in principle, but access is private in practice. This makes benchmark construction difficult and also entrenches illegibility by handicapping efforts at “open source” legal AI. Other AI domains provide evidence that open source promotes transparency, as they can usually be studied to a greater degree than their private equivalents (72).

Finally, for academics, benchmarking may come with decreasing marginal returns. Introducing the first benchmark or one for new tasks has novelty recognized by publication venues. But assessing newer systems on that benchmark, or making minor modifications to it, has limited additional research value, thus disincentivizing academic investments of time or resources.

#### 2.2.4. Governance bodies.

A final option would be for governmental bodies to lead benchmarking efforts. Existing regulators of legal practice (like bar associations or state supreme courts) might take a leading role here. State legislatures could also create novel state-level agencies to conduct benchmarking. Alternatively, benchmarking might be performed at the federal level, through NIST or another federal body.

A governmental approach to benchmarking is attractive because governments are uniquely positioned to represent public interests. Yet this approach faces substantial challenges across key design dimensions. Most notably, governments lack AI expertise and resources. Existing state regulators do not have the capacity to engage in benchmarking, particularly lacking the technical expertise necessary for sophisticated benchmark construction, evaluation protocol design, or assessment of subjective legal quality in their measurement choices. States, moreover, do not have the funding or resources to create novel agencies or regulatory bodies capable of comprehensive system evaluation. And even if the federal government was capable of marshaling technical expertise and funding through bodies like NIST, one might logically question its ability to develop benchmarks for state law.

Despite these limitations, governmental bodies would bring unique advantages. Their public interest mandate naturally supports appropriate task definition that represents diverse stakeholder needs rather than narrow commercial interests. Similarly, they would be well-positioned to ensure comprehensive system selection across market participants and maintain transparency that serves public rather than private interests. However, the technical capacity gaps mean that realizing these potential advantages would require substantial investment in expertise and infrastructure that current governmental institutions lack.

### 2.3. The Implications of Illegibility.

Illegibility can have several deleterious consequences for the adoption of legal AI. First, it stifles innovation. AI development is largely experimental, proceeding by trial-and-error. Information about how specific kinds of AI systems perform plays an essential role in allowing developers to improve design and construction. When information about performance is sparse, expensive, or inaccurate, making technical progress is challenging. Legal AI’s illegibility imposes a drag on innovation at multiple levels. Developers cannot learn from the failures of competing systems, nor can they easily identify the frontier of model performance or pinpoint persistent bottlenecks in existing tools. The absence of shared evaluation data or standardized benchmarks also inhibits cumulative progress. Researchers and engineers working across institutions can struggle to compare findings, validate one another’s claims, or build on prior work. As a result, efforts to develop new legal AI tools often proceed in isolation, with little visibility into what has or has not worked for others.

Second, illegibility creates a tension between tool utilization and adherence to professional obligations for lawyers. Ethical rules obligate competent representation, and an awareness of the benefits and risks associated with relied-upon technology (73). Lawyers thus need to understand how errors, such as hallucinations, might arise and the level of caution necessary with specific tools. Legal AI’s illegibility means this information is scarce, and lawyers cannot assess the appropriateness of reliance on a tool without understanding how that tool performs. For the same reasons, illegibility also impedes responsible procurement of legal technology by law firms.

Third, illegibility can hamper legal education. Law schools and bar associations are tasked with overseeing introductory and continuing forms of legal instruction. There are widespread calls for legal education to more directly grapple with the technology and effective AI usage (74). But institutions will struggle to fulfill this mission absent information about how tools actually function. The sheer prevalence of hallucinations in legal cases (10, 11) shows awareness is lacking.

Finally, illegibility hinders governance. The lack of detailed information about specific tools limits the kinds of guidance and rules that courts, bar associations, and legislatures can craft. This informational gap may help explain the widespread adoption of AI disclosure rules that require lawyers to disclose any use of AI (75). These mandates rely on a crude distinction between AI and non-AI tools—treating all uses of AI as equally significant—regardless of the system’s function or context (75). The absence of fine-grained performance information leaves policymakers little choice but to rely on such coarse proxies.

## 3. Potential Paths Forward

How might we address these institutional design challenges? Our analysis suggests that no single legal AI stakeholder possesses the combination of resources, expertise, and incentives necessary for trustworthy public benchmarking. This suggests a different approach: Rather than searching for an ideal institution to lead benchmarking, we should match institutional models to available resources and constraints.

This section examines three distinct approaches, each optimized for different budgets (high, medium, and low resource availability). For the high-resource setting, we suggest an independent and self-contained benchmarking effort styled after NIST’s FRVT. For the medium-resource setting, we recommend multi-institutional consortia styled after the legal track of the Text Retrieval Conference and MLPerf. And for the low-resource setting, we recommend targeted grants to mobilize specific benchmarking efforts. We show how each recommendation is informed by the institutional tradeoffs identified in Section 2.

We do not mean to suggest that these models exhaust the possibilities for addressing the institutional tradeoffs identified above. Another promising direction lies in carefully structured public–private partnerships. For instance, public governance bodies could collaborate with legal technology developers to certify that industry-led benchmarking efforts comply with established standards, thereby enhancing legitimacy and oversight. Such collaborative arrangements are closely related to our medium-resource proposal but warrant dedicated examination in future work.

### 3.1. The High Resource Setting.

In the high resource setting, we assume an institution has substantial financial resources, technical expertise, and legal expertise. One example is a committed nonprofit entity with the appropriate resources-like a large law-focused foundation. Prior to recent government cuts, a standard-setting agency like NIST would also have been a potential candidate, as it had AI talent and experience with legal benchmarking. Given sufficient resources, NIST could reemerge as a viable candidate.

In this setting, a benchmark can be conducted entirely in-house. The institution would decide which tasks to benchmark and evaluate a range of systems capturing different evaluation goals, such as systems with wide usage, innovative designs, or those created by open-source/academic efforts. It would repeat benchmarking regularly (e.g., annually) and publish results. It might also set up partnerships through which academics could partner with the organization to do specific research.

This institution would deliver the optimal results across Table 1’s rubric. Its independence allows the institution to pick tasks of broad interest (avoiding capture by legal technology consumers) and construct rigorous representative benchmarks (avoiding capture by developers). Its substantial resources and expertise also allow it to go beyond what academics and academic groups perform today.

NIST’s evaluation of facial recognition models offers an illustrative example. Since 1994, NIST has conducted periodic evaluations of different facial recognition technologies (76). These evaluations began with prototypes, and expanded to commercial systems as the technology matured, with vendors submitting systems for consideration. Reflecting the growth of the technology and developments in the evaluation science of facial recognition, benchmarking in each successive iteration has grown in breadth and complexity. The benchmark has shaped the market and allows potential users to assess performance on specific subgroups. Importantly, benchmarking is conducted on private data, limiting the ability of vendors to game the benchmark.

### 3.2. The Medium Resource Setting.

In the medium resource setting, we assume some financial resources and some expertise—approximately an order of magnitude less than what is available in the higher resource setting. Specifically, we assume an institution has the capacity to coordinate benchmarking, even if it cannot itself perform it.

Under these constraints, a potential approach is for this body to convene and coordinate a multi-institutional benchmarking consortium. This consortium would contain the different stakeholders identified in Section 2 (e.g., developers, consumers, academics, and oversight bodies). The logic underlying this form is to design a consortium which distributes decision-making across different actors according to their relative strengths and weaknesses. By pooling complementary expertise and assigning responsibility for different design dimensions to the institutions best positioned to handle them, consortia can overcome the deficits that have characterized prior, single-institution efforts.

Responsibilities might be sorted as follows: Public oversight bodies could play a leading role in selecting tasks. Academic researchers could establish procedures for benchmark construction and evaluation. Legal AI consumers would offer resources in terms of funding, and legal expertise in order to build the benchmarks and evaluate the outputs. And developers would participate by offering additional technical support where appropriate. Bar associations might offer Continuing Legal Education credit for lawyers who assist in the effort.

MLPerf serves as an inspiration for this model (77). In the late 2010s, a set of academic efforts sought to establish benchmarks for measuring the hardware performance of ML systems. Recognizing both the valuable role benchmarking plays and the lack of industry standards, MLPerf arose in 2018 as a collaboration between academic researchers and large industry companies to standardize performance measurement.

Although this institutional model can provide value, the reliance on private institutions comes with certain consequences. Law firms will only participate if the tasks and systems evaluated are relevant to their activities. Relative to the high resource setting, the tasks chosen might thus be more captured by these firms, and skewed toward representing their interests. This approach is only feasible if many firms participate, but the distributed nature of the effort might create standardization issues for the benchmark, resulting in lower benchmark quality. Participating law firms would also need a clear benefit to participating, which might result in “embargoes” on how information produced from benchmarking is published. Absent such measures, it would be possible for firms outside of the consortium to free-ride on the efforts of consortium members, thus disincentivizing participation. There are also questions as to how sustainable this approach may be over an extended period of time. As law firms grow more sophisticated with respect to AI–and possibly expand in-house talent–participation in the consortium presents diminishing returns.

### 3.3. The Low Resource Setting.

The final setting we consider is a low resource setting. Here, we assume resources an order of magnitude lower than the medium resource setting—an institution has limited funding and limited expertise.

An institution experiencing these constraints will be incapable of performing benchmarking itself or engaging in the type of coordination a consortium requires. Here, targeted research grants for narrowly scoped benchmarks may be the best path. Grants might be distributed to academic or nonprofit research entities, and fund either the creation of benchmarks or a benchmarking effort targeted at a small number of existing systems. For instances a grant could fund the creation of a benchmark for AI systems intended to classify the enforceability of lease provisions or the evaluation of the major legal search engines. The principal observation that emerges in this setting is that limited resources necessitates precision about which kinds of performance information are most useful. Put differently, the institutional coordinator must decide where legibility is most necessary or most likely to produce beneficial downstream impacts.

An important conclusion is that, in low-resource settings, benchmarking efforts should prioritize AI systems with access-to-justice implications. This strategic focus rests on several considerations. First, these systems primarily serve individuals who lack the means to evaluate the tools they use. While private firms retain some ability to produce basic performance information—and may eventually develop internal evaluation capabilities—ordinary users have far fewer resources, expertise, or institutional support to assess system quality. Second, the effectiveness of these tools has profound implications for the access-to-justice crisis. If consumer-facing legal AI systems perform poorly, their widespread use could harm vulnerable populations who depend on them for critical legal guidance. But if they prove effective, such tools could substantially expand access to legal assistance for millions of Americans unable to afford traditional representation.

Consider a concrete illustration: We currently lack reliable information about whether online chatbots like ChatGPT or Claude provide accurate legal advice, yet anecdotal reports suggest at least some users consult it for legal questions (78). A targeted benchmarking effort could develop focused evaluations across specific practice areas (e.g., bankruptcy, housing law, child custody disputes) where individuals most commonly seek assistance. Such benchmarks would provide essential information about when and how these widely accessible tools can be safely used.

To be clear, this strategy reflects the clear limitations of the low-resource setting. It essentially reflects a triage mentality, concentrating limited resources on the highest-stakes applications while ceding more comprehensive market-wide evaluation. Relying on academic research imports the limitations raised in Section 2 and limits the ability to raise awareness of risks. However, by focusing on areas where benchmarking gaps pose the greatest risks to vulnerable populations, this approach can still generate substantial public benefit even under severe resource constraints.

In this paper, we have considered legal AI’s legibility problem, the institutional design challenges of public benchmarking, and potential paths forward. Our emphasis is on tailoring benchmarks to available resources. Just as there is no free lunch, there is no free benchmark to resolve the tradeoffs we have identified.

## Data Availability

There are no data underlying this work.

## Footnotes

^*^In some domains, there is increasing use of automated performance measurements, but this remains difficult for legal AI and always requires human evaluation of validity and bias (39).

^†^In the context of legal AI specifically, Section 2 will describe illegibility’s different deleterious consequences.
